# Prophylactic digoxin treatment reduces IL-17 production *in vivo* in the neonatal calf and moderates RSV-associated disease

**DOI:** 10.1371/journal.pone.0214407

**Published:** 2019-03-25

**Authors:** Jodi L. McGill, Mariana Guerra-Maupome, Sarah Schneider

**Affiliations:** 1 Department of Veterinary Microbiology and Preventative Medicine, Iowa State University, Ames, Iowa, United States of America; 2 Department of Diagnostic Medicine and Pathobiology, Kansas State University, Manhattan, Kansas, United States of America; Kliniken der Stadt Köln gGmbH, GERMANY

## Abstract

Respiratory syncytial virus (RSV) is a leading cause of morbidity and mortality in human infants. Bovine RSV infection of neonatal calves is pathologically and immunologically similar to RSV infection in infants, and is therefore a useful preclinical model for testing novel therapeutics. Treatment of severe RSV bronchiolitis relies on supportive care and may include use of bronchodilators and inhaled or systemic corticosteroids. Interleukin-17A (IL-17) is an inflammatory cytokine that plays an important role in neutrophil recruitment and activation. IL-17 is increased in children and rodents with severe RSV infection; and in calves with severe BRSV infection. It is currently unclear if IL-17 and Th17 immunity is beneficial or detrimental to the host during RSV infection. Digoxin was recently identified to selectively inhibit IL-17 production by antagonizing its transcription factor, retinoid-related orphan receptor γ t (RORγt). Digoxin inhibits RORγt binding to IL-17 and Th17 associated genes, and suppresses IL-17 production *in vitro* in human and murine leukocytes and *in vivo* in rodent models of autoimmune disease. We demonstrate here that *in vitro* and *in vivo* digoxin treatment also inhibits IL-17 production by bovine leukocytes. To determine the role of IL-17 in primary RSV infection, calves were treated prophylactically with digoxin and infected with BRSV. Digoxin treated calves demonstrated reduced signs of clinical illness after BRSV infection, and reduced lung pathology compared to untreated control calves. Digoxin treatment did not adversely affect virus shedding or lung viral burden, but had a significant impact on pulmonary inflammatory cytokine expression on day 10 post infection. Together, our results suggest that exacerbated expression of IL-17 has a negative impact on RSV disease, and that development of specific therapies targeting Th17 immunity may be a promising strategy to improve disease outcome during severe RSV infection.

## Introduction

Human respiratory syncytial virus (HRSV) is a leading cause of severe acute lower respiratory tract disease in infants and young children worldwide [1, 2] and accounts for up to 70% of hospitalized bronchiolitis cases in industrialized countries [3, 4]. Globally, there are an estimated 33 million new episodes of HRSV-associated disease in children under five years of age with more than 100,000 resultant deaths [5].

Bovine respiratory syncytial virus (BRSV) is genetically and antigenically closely related to HRSV and is a primary cause of severe acute lower respiratory tract disease in young cattle. Following outbreaks of bovine respiratory disease, rates of seroconversion to BRSV can reach up to 45% [6]. In the instance of enzootic calf pneumonia, BRSV is a documented cause in as many as 60% of the cases [7]. BRSV infection in cattle is clinically, immunologically and histologically similar to RSV infection in humans [8–10], and thus is an opportunity to model the disease using a naturally-susceptible host-pathogen interaction.

IL-17 is a pro-inflammatory cytokine that is critical for neutrophil recruitment and activation [11]. It has been implicated in the pathogenesis of several autoimmune and chronic inflammatory diseases. Extensive research has been aimed at identifying therapeutic agents that will specifically interfere with IL-17 production and modulate its downstream inflammatory effects. Retinoid-related orphan receptor γ t (RORγt) is the transcription factor responsible for regulation of Th17 CD4 T cells [12] and IL-17 expression by activated neutrophils and γδ T cells [13, 14]. Digoxin is a small molecule that has recently been shown to specifically antagonize activity of RORγt and potently inhibit IL-17 production in a mouse model of multiple sclerosis [15]. Digoxin treatment is specific for the IL-17 pathway and does not alter the responses of other T helper subsets, or production of IL-2 or IFNγ [15]. Digoxin-mediated inhibition of RORγt and IL-17 production was shown to be highly efficacious in delaying and reducing the severity of the autoimmune disease [15]. These results have subsequently been confirmed in other *in vitro* and *in vivo* rodent models [16–22], and *in vitro* using human peripheral blood mononuclear cells (PBMC) [15]. Digoxin is a cardiac glycoside that is used in humans and veterinary species, including cattle, for the treatment of congestive heart failure or chronic respiratory disease. It has long been described to have anti-inflammatory properties [23], even in the face of bacterial pneumonia [24, 25]. These recent reports shed new light on the mechanism of digoxin’s function and the role of IL-17 in inflammation.

Calves and human infants with severe RSV disease display bronchointerstitial pneumonia and necrotizing bronchiolitis, with significant neutrophil infiltration of the airways, epithelial sloughing and mucus production [8–10]. The majority of the tissue damage observed during RSV infection is a result of the host inflammatory response [26–29] with recruitment and activation of neutrophils thought to be a primary cause of immunopathology. IL-17 is significantly increased in the lungs of mice and children infected with RSV [30–39]. Our own published work has shown that IL-17 is also induced in the lungs of calves infected with BRSV [40]. It is currently unclear if IL-17 plays a beneficial or detrimental role during RSV infection in the infant. Some studies in human infants have reported a negative correlation between IL-17 production and severity of RSV infection [30, 34, 39], and a study using neonatal mice has shown that treatment with recombinant IL-17 results in reduced lung inflammation during acute RSV infection [32]. Contrasting reports have shown significantly increased levels of IL-17 in tracheal aspirates from severely ill, ventilated infants compared to healthy controls [35, 37, 38]; and mice deficient in IL-17 display reduced mucus production and increased CD8 T cell responses in the lungs during primary RSV infection [35].

Due to the many similarities in disease pathogenesis and immunity, the calf model of BRSV is ideally suited for clarifying the role of IL-17 during RSV infection. Here, calves were administered digoxin to inhibit IL-17 production, and then infected with BRSV. Calves treated with digoxin demonstrated reduced virus-associated clinical signs during the late stages of infection, reduced gross lung pathology, and alterations in the inflammatory cytokine profile on day 10 post infection compared to untreated control calves. Interestingly, calves treated with digoxin also developed higher titers of IgA in nasal secretions by day 10 post infection compared to untreated animals. Although digoxin treatment reduced pulmonary IL-17 expression, it was not completely abrogated. Thus, our results demonstrate that exacerbated Th17 immunity likely contributes to disease pathogenesis in the late stages of RSV infection; however, it remains unclear if some amount of IL-17 may play a beneficial role in host protection.

## Materials and methods

### Animals

All animal procedures were conducted in strict accordance with federal and institutional guidelines and were approved by the Kansas State University Institutional Animal Care and Use Committee.

For initial *in vitro* digoxin studies, peripheral blood was collected from eight female adult Holstein cows that were housed at the Kansas State University Dairy in Manhattan, KS. Animals were group housed in outdoor pens, with ab libitum access to food and water.

For the *in vivo* digoxin/BRSV challenge study: thirty-two, colostrum replete, mixed-gender Holstein calves were enrolled at 1–2 weeks of age and were randomly assigned to four treatment groups (8 animals/group): no digoxin treatment, no BRSV infection; digoxin treatment, no BRSV infection; no digoxin treatment, BRSV infection; digoxin treatment, BRSV infection. Calves were housed by treatment group under environmentally controlled, BSL2Ag conditions in the Large Animal Research Facility at Kansas State University. Animals were fed milk replacer by nipple bottle twice per day, with ab libitum access to starter grain and water. The animal care protocol included provisions for a humane endpoint as determined by the discretion of the attending clinical veterinarian. Methods to minimize pain and distress included the avoidance of prolonged restraint and the inclusion of euthanasia as an intervention strategy.

**Fig 1:**
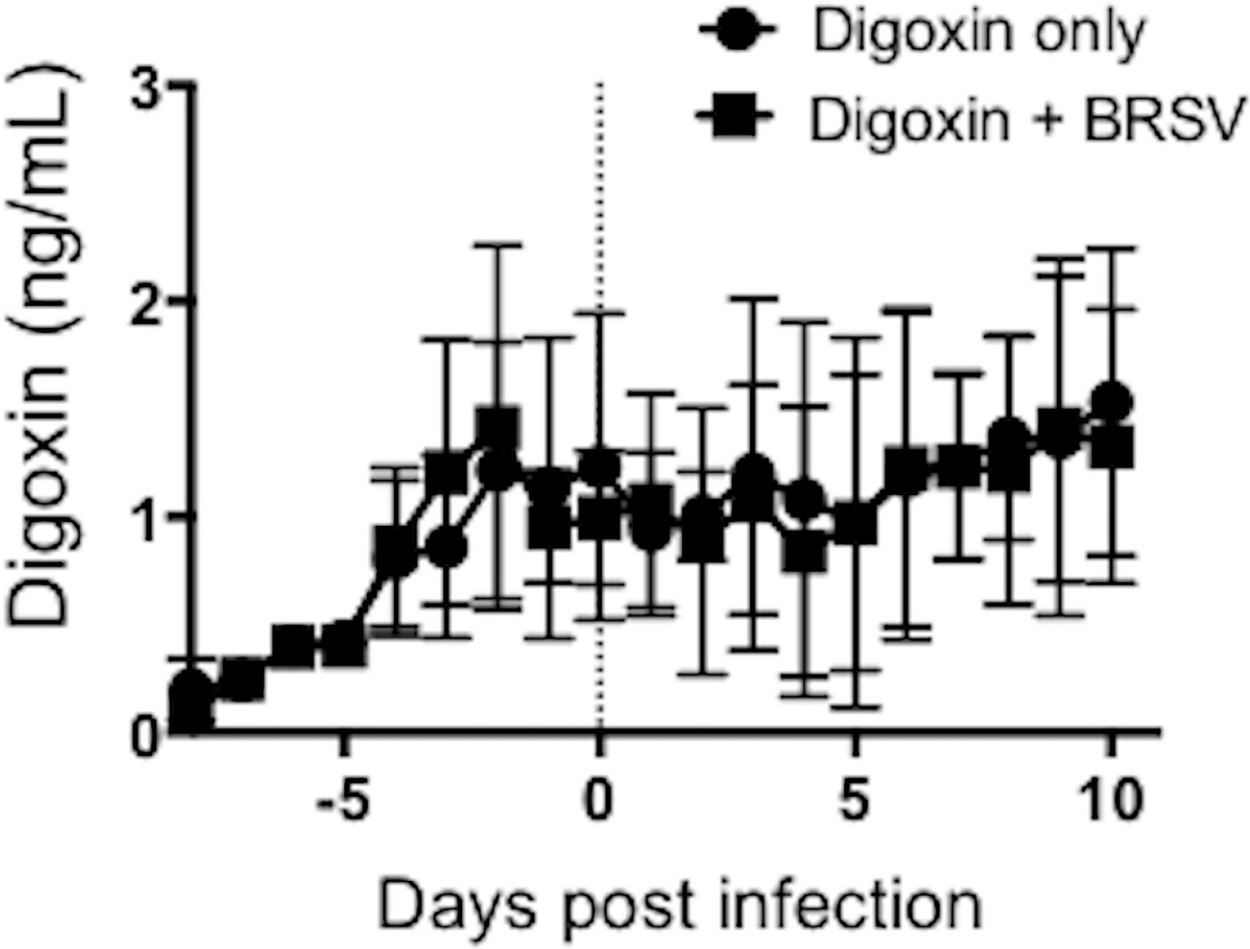
Circulating digoxin concentrations in calves receiving twice daily oral digoxin treatment. Treatments include: digoxin without BRSV infection (n = 8); digoxin with BRSV infection (n = 8); no digoxin treatment, no BRSV infection (n = 8) and no digoxin treatment with BRSV infection (n = 8). Calves in digoxin treated groups received the drug twice per day, administered in the milk replacer. Peripheral blood was collected once daily, at the same time each day, and serum samples were analyzed for circulating digoxin concentrations using a commercial ELISA kit. Treatment was initiated at a dose of 5 μg/kg/feeding, then increased over a period of several days until the desired therapeutic range of 0.8–2 ng/mL was achieved. A final dose of 80 μg/kg/feeding was selected 72 hours prior to BRSV infection (day -3). Calves were challenged via aerosol inoculation with BRSV strain 375 (day 0). Peripheral blood digoxin concentrations continued to be monitored daily through day 10 post infection, when the study was terminated. Data represent means ± SEM.

The first several days of the study were used to optimize the digoxin dosing regimen. Digoxin was administered orally, twice per day, suspended in the milk replacer. Peripheral blood was collected once daily, two hours after the first feeding of the morning, for evaluation of circulating digoxin levels. Digoxin levels in serum were determined using the Digoxin Accubind commercial ELISA kit (Monobind Inc.). The ideal therapeutic range for digoxin is estimated between 0.8–2 ng/mL (Merck Manuals and Merck Veterinary Manuals). While a few historical studies are available to suggest an appropriate dosing scheme for digoxin in bovines [41, 42], experiments were conducted in adult ruminants, and thus were difficult to extrapolate to pre-ruminant calves. Therefore, calves initially received a dose of 5 μg/kg/feeding, which was incrementally increased until the desired therapeutic range was achieved. A dose of 80 μg/kg/feeding was selected. As seen in Fig 1, calves receiving this dose had a mean circulating digoxin concentration of 1.2 ± 0.29 ng/mL digoxin on day 0 of the study, and this concentration was maintained for the duration of the study. Calves were observed several times daily and developed no signs of digoxin toxicity at these dosing levels, and no tachycardia, arrhythmias, or changes in pulse that could be attributed to the drug.

Peripheral blood was collected daily for measurement of digoxin levels and samples were banked to measure serum antibody titers. Blood was collected for measurements of the cellular immune response at two time points: once at 4 days prior to challenge to measure mitogen-activated cytokine production, and again on day 10 post infection to measure virus-specific cytokine production. Nasal swabs were collected on days 3, 6 and 10 for analysis of virus shedding. Nasal fluid was collected on days 6 and 10 post infection, as previously described [43], for measurement of virus-specific IgA production.

### BRSV inoculum and aerosol challenge model

BRSV strain 375 was prepared from virus stock re-isolated from the lung of an infected animal and passaged less than 4 times on bovine turbinate (BT) cells. The viral inoculum was determined free of contaminating BVDV by PCR. Calves were inoculated via aerosol challenge with ~10^4^ TCID_50_/mL of BRSV strain 375 as previously described [44].

### Antigen recall assays

Peripheral blood mononuclear cells (PBMC) were prepared as previously described [40, 45]. 5x10^6^ cells/mL were resuspended in cRPMI and plated in round-bottom 96-well plates. Bronchoalveolar lavage fluid (BAL) samples were filtered through 2-layer sterile gauze and centrifuged 500g for 10 minutes at 4°C. 2x10^6^ cells/mL were resuspended in cRPMI and plated in 24-well plates. Cells were stimulated with 0.01 MOI of BRSV. Negative control wells were stimulated with media only; positive control wells were stimulated with 1 μg/mL pokeweed mitogen. Plates were incubated for 6 days (PBMC) or 72 hours (BAL) at 37° C in a 5% CO_2_ incubator. Cell culture supernatants were collected and stored at -80° C for ELISA analyses.

### Clinical illness scoring

Calves were scored once daily for clinical illness by a trained, blinded observer. Calves were scored using an adaptation of the University of Wisconsin Calf Health Respiratory Scoring Chart, originally established by Dr. Sheila McGuirk (https://www.vetmed.wisc.edu/dms/fapm/fapmtools/8calf/calf_respiratory_scoring_chart.pdf). The scoring chart assigns numbers (0–3) based upon fever and severity of clinical signs that include cough, nasal discharge, eye crusting and ear position. For our scoring chart we include one additional category for expiratory effort (0 = no effort to 3 = significant effort). The scores for each category are totaled to determine the overall clinical score, with a maximum possible score of 18.

### Necropsy and pathological evaluation

Calves were euthanized on day 10 post-infection by barbiturate overdose (sodium pentobarbitol, administration of 100 mg/kg or dose to effect). Pathological evaluation was performed similar to previous descriptions [44, 46]. The extent of pneumonic consolidation was evaluated using the scoring system we have previously described [43].

BAL was collected by introducing 500 mL of sterile, ice-cold PBS through the trachea. Samples of affected and unaffected lungs were collected from multiple sites for histopathological evaluation. Tissues were fixed by immersion in 10% neutral buffered formalin and processed by routine paraffin-embedment and sectioning. 5 μM sections were stained for hematoxylin and eosin. Microscopic lesions were evaluated by a pathologist (Dr. Schneider) in a blinded manner. The severity of the lung lesions were scored based upon the criteria we have previously described [43].

BAL samples were cryopreserved in 10% DMSO and FBS and stored in liquid nitrogen. Cells were then thawed and washed twice in cRPMI, then cells were adjusted to 2x10^5^ viable cells/mL in cRPMI. Samples were submitted to the Iowa State University Veterinary Diagnostic Laboratory for preparation of cytospins and differential staining (Modified Wrights Stain). Cytology was performed by a blinded individual and a minimum of 500 cells were counted per sample. Duplicate samples were prepared for each animal.

### Real-time PCR

RNA isolation, cDNA preparation and qPCR were performed as described [40, 45]. The primer sequences have been published [40, 44]. Relative gene expression was determined using the 2^−ΔΔCt^ method [47], with RPS9 as the reference housekeeping gene.

NS2 copy number was quantified as we have previously described [43]. Briefly, lung samples from representative lesioned and non-lesioned tissues from each calf were collected and stored in RNAlater. RNA was isolated from lung tissue samples using Trizol Reagent (Life Technologies). Total RNA was quantified by Nanodrop and 500 ng of total RNA were used in each reaction. cDNA synthesis and quantitative rtPCR reactions were carried out using the Taqman RNA-to-CT 1-step kit (Applied Biosystems) per manufacturer’s instructions. Primers and probes for the BRSV NS2 gene and the bovine S9 gene have been published [43]. The reactions were performed on an Agilent MX3000P Real-Time PCR machine with the following cycling conditions: 48° C hold for 15 minutes; 95° C hold for 10 minutes; 40 cycles of 95° C for 15 s, then 60°C for 1 minute. Standard curves for NS2 and S9 genes were run in parallel with test samples, and all standards and test samples were run in triplicate. Viral NS2 copy numbers were calculated using standard curves and normalized to S9 to correct for differences in input material.

### Virus isolation

Nasal swabs were collected from each calf on days 0, 3 and 6 and 9 post infection and placed in virus transfer media. Virus isolations were performed as previously described [44].

### ELISAs

Bovine IL-17A and IFNγ VetSet ELISA Development kits were purchased from Kingfisher Biotech, Inc and performed according to manufacturer’s instructions.

Indirect ELISAs were performed as previously described [43] to quantify IgA in the nasal and BAL fluid, and IgG in the serum. ELISA plates were coated overnight at 4° C with 100 μl/well of BRSV stock (~10^4^ TCID_50_), or with 3μg/mL recombinant F or G protein. Negative control wells were coated with 100 μl/well cell culture media prepared from uninfected BT. Nasal fluid samples were treated with 10 mM dithiothreitol (DTT) for 1 hour at 37° C prior to performing the ELISAs. Serum samples were not treated. Samples were plated in duplicates, incubated for 2 hours at room temperature and then washed. Mouse anti-bovine IgA-HRP (Bethyl Laboratories) or anti-bovine total IgG-HRP (Bethyl Laboratories) was used at 0.5 μg/mL. Plates were developed using Pierce 1-Step Ultra TMP Substrate (ThermoScientific Pierce). The reactions were stopped with the addition of 0.2 M H_2_SO_4_ and plates were read at an optical density of 450 nm and 540 nm using an automated plate reader.

### Virus neutralizing activity

Samples of nasal fluid and serum were submitted to the Kansas State University Veterinary Diagnostic Laboratory for determination of neutralizing antibody titers.

### Statistics

Statistical analysis was performed using Prism v6.0h software (Graphpad Software, Inc.). The data were analyzed using a Kruskal-Wallis test followed by Dunn’s Multiple Comparison’s test. Clinical data were analyzed by two-way ANOVA with repeated measures, followed by Sidak’s multiple comparisons test.

## Results

### *In vitro* and *in vivo* digoxin treatment inhibits IL-17 production by bovine PBMC

**Fig 2:**
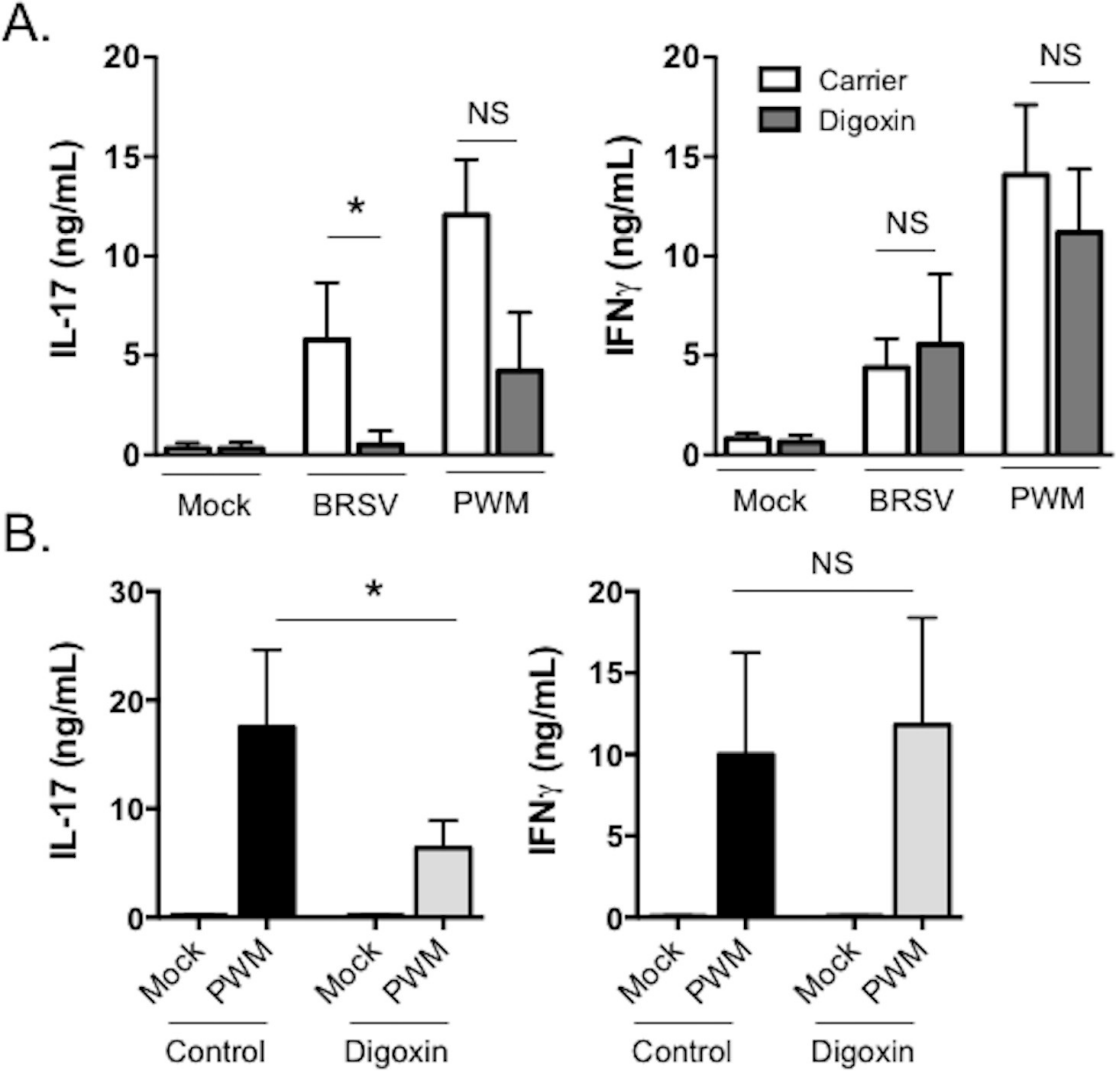
*In vitro and in vivo* digoxin treatment inhibits IL-17, but not IFNγ secretion, by bovine PBMC. **(A)** Peripheral blood was collected from adult, BRSV-immune cows (n = 8) housed at the Kansas State University Dairy. PBMC were isolated and plated at 2x10^5^ cells per well in a 96-well plate. Cells were stimulated for 6 days with a 0.01 MOI of BRSV strain 375, or 1 μg/mL pokeweed mitogen (PWM) in the presence of 2 μM digoxin (digoxin) or DMSO (carrier control). Unstimulated cells (mock) served as negative control wells. On day 6, cell culture supernatants were harvested and the concentrations of IL-17 and IFNγ were determined by commercial ELISA kits. **(B)** PBMC were isolated from control and digoxin treated calves (n = 16, as outlined in Fig 1) on day -3 prior to BRSV infection. Cells were stimulated for 6 days with 1 μg/mL pokeweed mitogen (PWM). Unstimulated cells (mock) served as negative control wells. After 6 days, cell cultures supernatants were analyzed by ELISA, as in (A). Data represent means ± SEM. *p<0.05 as determined by Kruskal-Wallis test, followed by Dunn’s multiple comparisons test. (NS) no significant differences were observed.

Digoxin specifically inhibits IL-17 production *in vitro* by murine and human peripheral blood cells, and *in vivo* when administered to mice and rats [15–18]. In preliminary experiments we tested the capacity of digoxin to prevent IL-17 production by bovine peripheral blood cells. Bovine PBMC were stimulated for 6 days with BRSV strain 375 or pokeweed mitogen in the presence or absence of 2 μM digoxin or carrier control (DMSO). The concentration of digoxin was selected based upon published studies with murine and human PBMC [15–18]. Cell culture supernatants were analyzed for concentrations of IL-17 and IFNγ using a commercial ELISA kit. As seen in Fig 2A, digoxin significantly inhibited the secretion of IL-17 by PBMC stimulated with BRSV. Bovine PBMC stimulated with pokeweed mitogen in the presence of digoxin also secreted reduced levels of IL-17, although this reduction was not statistically significant. Consistent with previous reports using human and murine cells [15–18], IFNγ secretion was not affected by digoxin treatment (Fig 2A).

We next determined if *in vivo* digoxin treatment had the capacity to limit IL-17 production by bovine PBMC. A total of 32 calves were divided into four groups of n = 8 animals each. Two groups received oral digoxin treatment, two groups did not (Fig 1). On day -2, PBMC were isolated and stimulated with pokeweed mitogen. After 6 days, cell culture supernatants were collected and analyzed for IL-17 and IFNγ concentrations by commercial ELISA kit. As seen in Fig 2B, *in vivo* digoxin treatment significantly inhibited IL-17 secretion in response to mitogen treatment, but did not impact the capacity of the cells to secrete IFNγ. Together, these results confirm reports in rodent species, showing that digoxin has the capacity to specifically inhibit IL-17 production by bovine immune cells, without negatively impacting IFNγ production.

### Therapeutic inhibition of IL-17 production reduces RSV-associated disease

**Fig 3:**
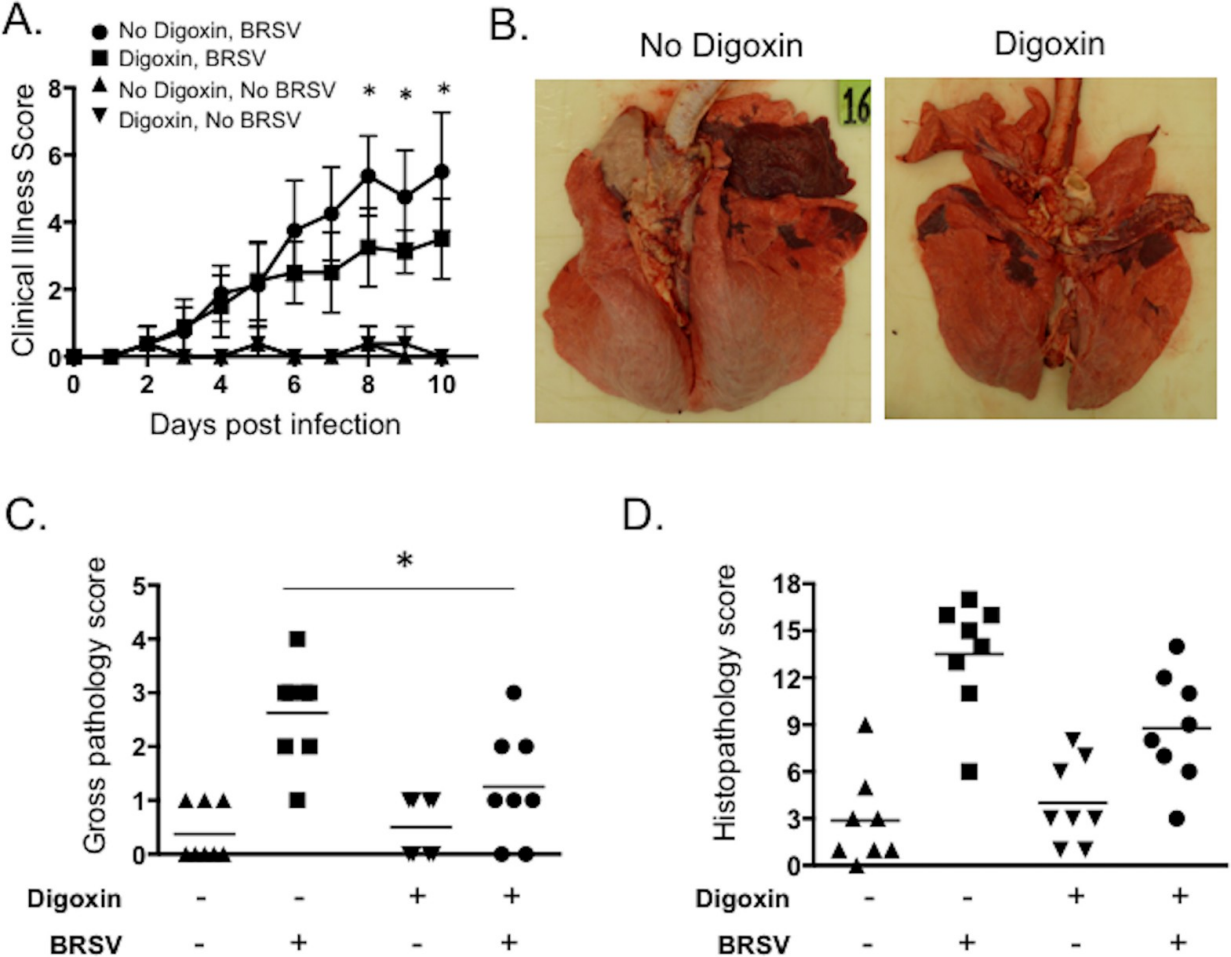
Therapeutic inhibition of IL-17 production reduces RSV-associated disease in neonatal calves. Once therapeutic levels of digoxin were established (Fig 1), calves were challenged with BRSV strain 375 via aerosol inoculation. (**A**) Calves in all four groups were monitored daily by a blinded observer and assigned a clinical score using the criteria outlined in Materials and Methods. Data represent means ± SEM. *p<0.05 as determined by 2-way ANOVA with repeated measures, followed by Sidak’s multiple comparisons test. (**B-D**) Animals were humanely euthanized on day 10 post infection. The extent of gross pneumonic consolidation was evaluated based upon the percent of lung affected (0 = free of lesions; 1 = 1–5% affected; 2 = 5–15% affected; 3 = 15–30% affected; 4 = 30–50% affected; 5 = >50% affected). Representative images of the lungs from 2 BRSV infected calves are shown in (**B**). The animal on the left did not receive digoxin. The animal on the right was treated with digoxin. Aggregate gross pathology results from all groups and all animals are depicted in (**C**). *p<0.05 as determined by Kruskal-Wallis test, followed by Dunn’s multiple comparisons test. Sections of lung were collected from multiple locations and microscopic lesions were evaluated by a pathologist in a blinded manner using a scoring system we have previously described (see Materials and Methods). Aggregate histopathology scores from all animals are depicted in (**D**). No significant differences were observed between treatment groups.

Once therapeutic levels of digoxin had been maintained for 72 hours, one group of digoxin-treated calves and one group of untreated calves were challenged via aerosol inoculation with BRSV Strain 375 as we have previously described [8, 40, 43, 45, 48]. The remaining animals (with or without digoxin treatment) served as uninfected controls. All calves were monitored daily for clinical signs, including fever and respiratory symptoms, using the scoring system outlined in the Materials and Methods. Animals in both the digoxin treated and untreated groups developed elevated temperatures. Mild clinical signs were also observed in both challenged groups, including cough, increased expiratory effort and nasal discharge; and were apparent starting on days 4–6 post infection. As seen in Fig 3A, there were no differences in clinical signs between the digoxin treated and untreated calves in the first several days after infection. However, on days 8–10 post infection, the digoxin treated calves exhibited significantly reduced clinical signs compared to control, untreated calves (Fig 3A).

The animals were euthanized on day 10 post infection and a necropsy was performed. Calves challenged with BRSV demonstrated regional focal to coalescing lung consolidation, consistent with our previous studies [43, 44]. As see in Fig 3B and 3C, calves treated with digoxin demonstrated significantly reduced gross lung pathology on day 10 post infection compared to untreated, BRSV infected control calves. A few calves in the uninfected control groups (with and without digoxin treatment) demonstrated minor, focal areas of consolidation (Fig 3C). The cause of these lesions in the control animals is unknown, as samples submitted for diagnostic testing were negative for the common pathogens associated with bovine respiratory disease. All calves received a prophylactic antibiotic prior to enrollment in the study.

Lung samples from all calves were evaluated for microscopic lesions using the scoring system we have previously described [43]. As seen in Fig 3D, although there is a trend towards a reduction in microscopic lung lesions in the digoxin treated, BRSV-infected calves, the differences were not statistically significant between groups.

### Therapeutic inhibition of IL-17 production does not impact BRSV lung burden or shedding

**Fig 4:**
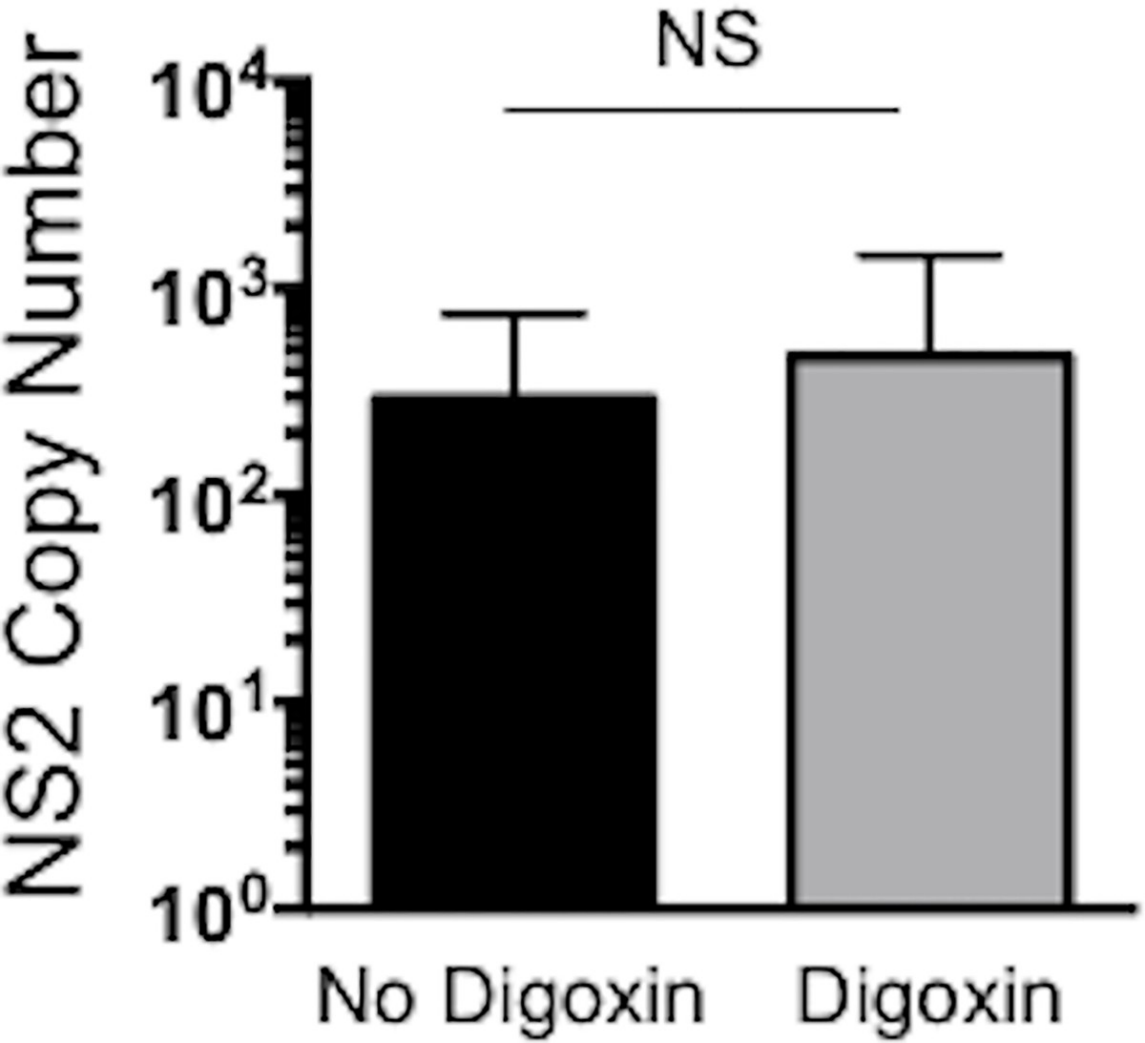
Digoxin treatment has no impact on lung viral burden. Lung samples were collected from representative lesion and non-lesion sites of the lungs on day 10 post infection and preserved in RNALater. Tissues samples were evaluated for viral burden by qPCR for the BRSV NS2 gene. Viral NS2 copy numbers were calculated using standard curves and normalized to the housekeeping gene, S9, to correct for differences in input material. Data points represent the means ± SEM of each BRSV-infected group. Lung samples from uninfected control calves were negative for the BRSV NS2 gene and were not depicted on the graph. (NS) No significant differences were observed between digoxin treated and untreated groups.

BRSV was isolated with similar frequency from nasal swabs collected from both digoxin treated and untreated calves on days 3 and 6 post infection (Table 1). Only one calf in each group was still shedding virus on day 10 post infection. As seen in Fig 4, lung viral burdens were minimal in both groups on day 10 post infection, and not significantly different between digoxin treated and untreated calves. BRSV was not detected in the lungs of the uninfected control calves, regardless of digoxin treatment.

**Table 1:** Virus isolation results from nasal swabs collected on day 0, 3, 6 and 10 after infection.

Treatment groups		Days post infection			
		Day 0	Day 3	Day 6	Day 10
Group 1	No digoxin, no BRSV infection	0/8	0/8	0/8	0/8
Group 2	Digoxin, no BRSV infection	0/8	0/8	0/8	0/8
Group 3	No digoxin, BRSV infection	0/8	7/8	8/8	1/8
Group 4	Digoxin, BRSV infection	0/8	7/8	8/8	1/8

### Digoxin treatment reduces *in vivo* expression of IL-17 and Th17 associated cytokines and alters BAL leukocyte populations during BRSV infection

**Fig 5:**
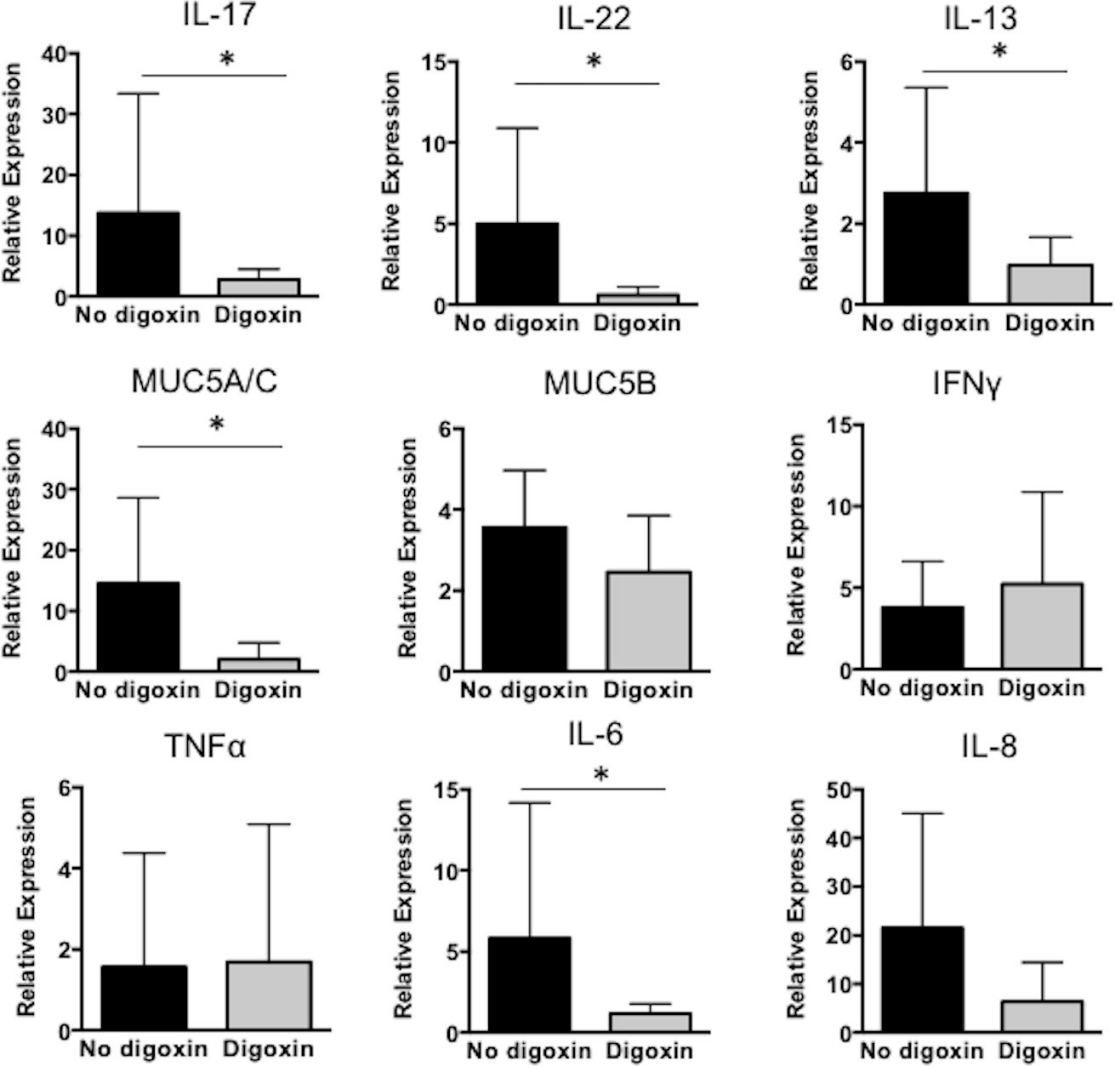
Digoxin treatment alters inflammatory cytokine expression in the lungs during BRSV infection. Lung tissues samples from all calves were analyzed by qPCR for mRNA expression of IL-17, IL-22, IL-13, MUC5A/C, MUC5B, IFNγ, TNFα, IL-6 and IL-8. Results were normalized to the housekeeping gene RPS9, and expressed relative to the uninfected, untreated control calves. Data represent means ± SEM. *p<0.05 as determined by Kruskal-Wallis test, followed by Dunn’s multiple comparisons test.

Lung samples were collected from digoxin-treated and untreated calves and analyzed by qPCR for mRNA expression of IL-17 and the Th17 associated cytokine IL-22 on day 10 post-BRSV infection. Digoxin-treated calves demonstrated reduced expression of both cytokines (Fig 5). Calves treated with digoxin also expressed lower levels of MUC5A/C, IL-13 and IL-6 in the lungs compared to untreated, BRSV-infected calves. However, we observed no significant differences in the expression of IFNγ, TNFα or IL-8 expression between digoxin treated and untreated control calves at this time (Fig 5).

**Fig 6:**
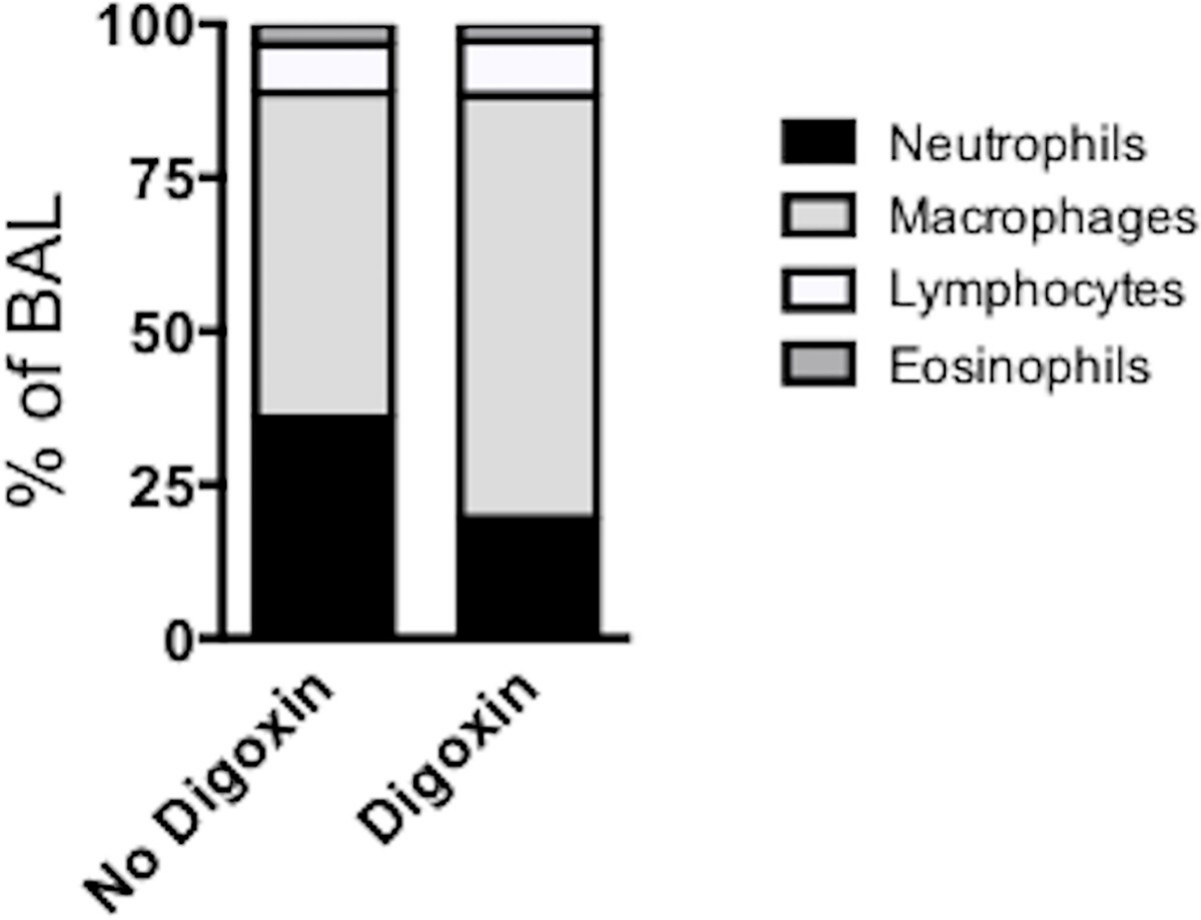
Altered leukocyte profiles in the BAL of digoxin treated calves. BAL samples were collected from all BRSV infected calves (with and without digoxin treatment) on day 10 post infection and cryopreserved in liquid nitrogen. Samples were thawed and the cell concentration was adjusted to 2x10^5^ cells/mL. Cytopin preparations were made and cells were differentially stained with Modified Wright Stain. Numbers of neutrophils, macrophages, lymphocytes and eosinophils were determined by microscopy. Data are depicted as the mean relative frequencies of each population.

The significant alterations in lung cytokine expression between digoxin treated and control calves suggested that we may also observe changes in the profile of inflammatory cells that were recruited to the lung during infection. Cytospin preparations of the BAL were differentially stained and the relative numbers of neutrophils, macrophages, lymphocytes and eosinophils were determined by microscopy. As seen in Fig 6, compared to untreated controls, digoxin treated calves had reduced relative frequencies of neutrophils, and increased frequencies of macrophages in the BAL on day 10 post infection (*p<0.05). There were no significant differences in lymphocyte or eosinophil frequencies in the BAL at this time.

### Therapeutic inhibition of IL-17 positively impacts virus-specific IgA production

**Fig 7:**
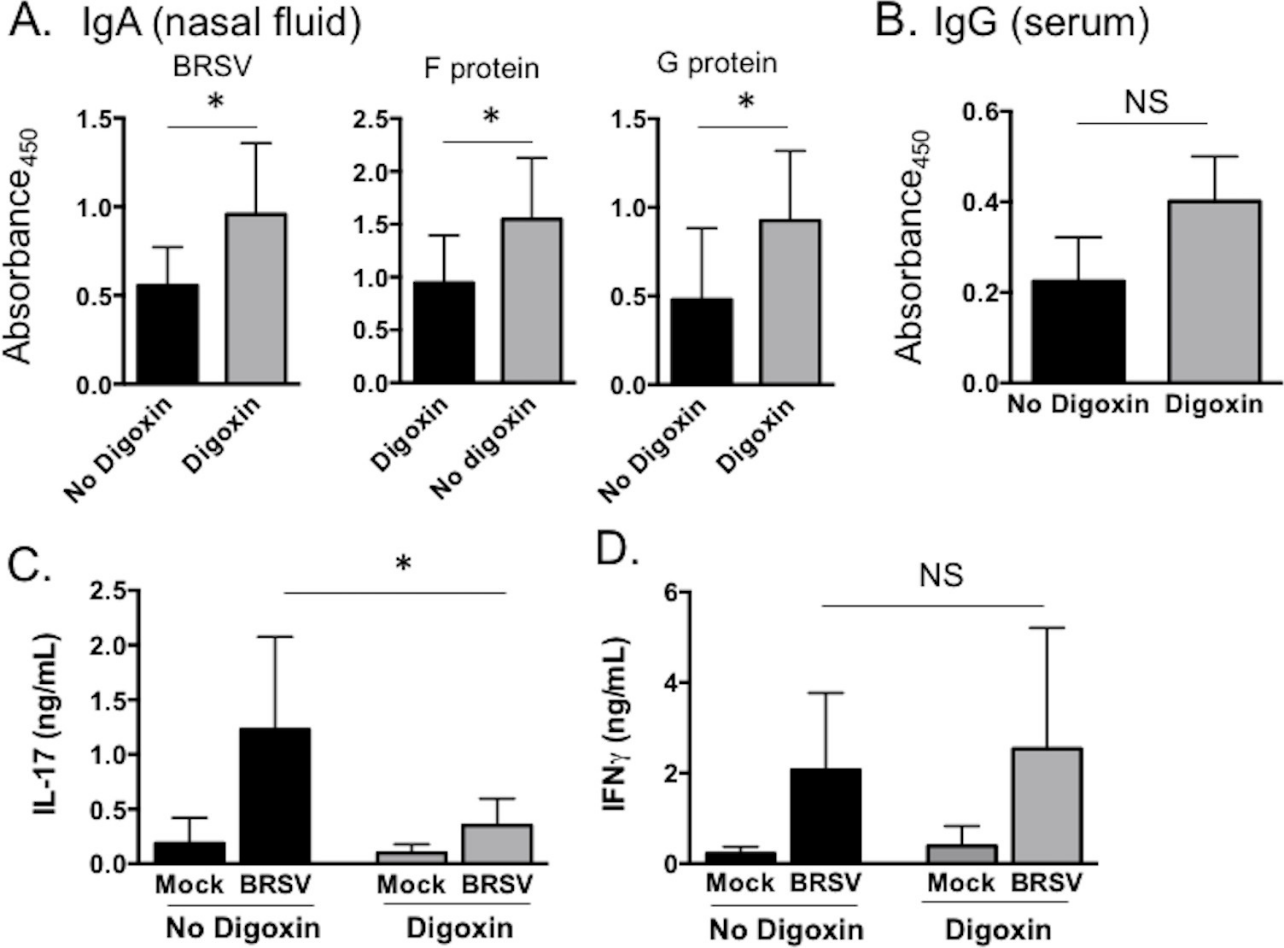
Therapeutic inhibition of IL-17 production alters the mucosal immune response to BRSV infection. (**A**) Nasal fluid samples were collected on day 10 post infection and analyzed for BRSV-specific IgA by indirect ELISA. (**B**). Serum samples were collected on day 10 post infection and analyzed for BRSV-specific IgG by indirect ELISA. **(C** and **D**) Bronchoalveolar lavage samples were collected during the necropsy on day 10 post infection. BAL cells were stimulated for 72 hours with BRSV, or remained unstimulated, as in B. Cell culture supernatants were collected and concentrations of (**C**) IL-17 and (**D**) IFNγ were measured by commercial sandwich ELISA. Data represent means ± SEM. *p<0.05 as determined by Kruskal-Wallis test, followed by Dunn’s multiple comparisons test.

Nasal fluid and serum samples were analyzed for virus-specific IgA and IgG, respectively. Virus-specific IgA was undetectable in either group on day 6 post infection, and was below the limit of detection in uninfected control calves, regardless of digoxin treatment. However, by day 10 post infection, digoxin treated calves had significantly higher levels of virus-specific IgA in the nasal fluid compared to untreated calves (Fig 7A). The IgA response in the nasal fluid was specific to both the F and G proteins from BRSV (Fig 7A), and we observed no significant differences in the IgA specificity as a result of digoxin treatment. Consistent with the ELISA results, neutralizing antibody titers in nasal fluid were also higher in the digoxin treated calves compared to the control animals (Table 2). Virus-specific IgG levels were not significantly different between digoxin treated and untreated groups on day 10 post infection (Fig 7B). Similarly, neutralizing serum antibody titers were not different between groups (Table 2). BRSV-specific IgG responses do not develop until several weeks after infection [49], and the calves used in this study were colostrum-replete. Therefore, the BRSV-specific IgG measured in the serum is likely attributable to maternally-derived antibody.

**Table 2:** Neutralizing antibody titers in nasal fluid and serum on day 10 post infection.

Treatment Groups		Nasal Fluid	Serum
Group 1	No digoxin, no BRSV infection	3.4 (2–8)	10.9 (4–32)
Group 2	Digoxin, no BRSV infection	3.7 (2–8)	8 (4–16)
Group 3	No digoxin, BRSV infection	29 (16–64)	10.3 (4–32)
Group 4	Digoxin, BRSV infection	52 (32–64)	16 (8–32)

Virus-specific cellular immune responses were measured in the bronchoalveolar lavage. BAL cells were isolated on day 10 post infection and were restimulated for 72 hours with BRSV as in Fig 1. Cell culture supernatants were then analyzed for concentrations of IL-17 (Fig 6C) and IFNγ (Fig 6D) using a commercial ELISA kit. We observed significantly reduced concentrations of virus-specific IL-17 in the BAL cell culture supernatants from BRSV infected, digoxin treated calves compared to untreated, infected control calves, but no difference in the concentration of IFNγ.

### IL-17 and Th17 immunity play a central role in shaping the inflammatory response to BRSV infection

**Fig 8:**
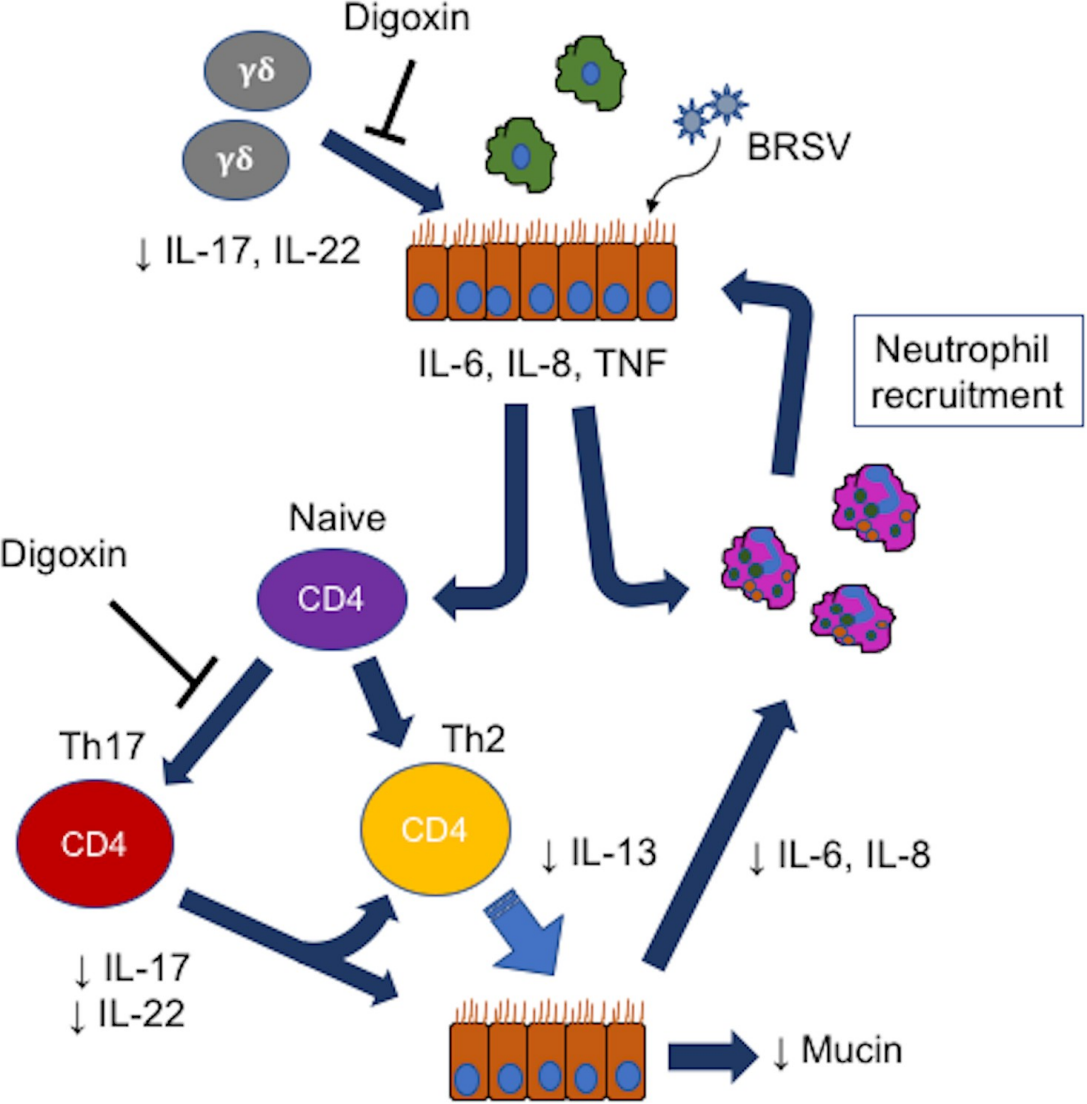
Proposed role of IL-17 during BRSV infection and mechanisms of action by therapeutic digoxin treatment. Therapeutic treatment with digoxin impacts several downstream inflammatory processes, including Th1/2/17 cell differentiation, IL-6 and IL-8 secretion, mucin production and neutrophil recruitment to the lungs.

Our data suggest that reducing production of IL-17 during acute BRSV infection has significant downstream effects on the inflammatory milieu in the lungs and the subsequent adaptive immune response. Based on our results, we have proposed a model by which IL-17, and digoxin treatment, are influencing the immune response to BRSV in the calf (Fig 8). CD4 and γδ T cells use the RORγt transcription factor to regulate their expression of IL-17 and IL-22 [11]; therefore, digoxin has the capacity to target both cell types. As seen in Fig 8., initial BRSV infection induces expression of early inflammatory mediators such as IL-6, IL8 and TNFα. These signals help initiate neutrophil and immune cell recruitment, and begin the process of CD4 T cell activation and differentiation into Th17 cells. BRSV infection also activates γδ T cells which are present in the lung and can serve as a source of IL-17 and IL-22. Prophylactic digoxin treatment targets and reduces early IL-17 production by γδ T cells and reduces CD4 Th17 differentiation. IL-17 acts on lung stromal cells to produce IL-8, thus indirectly promoting neutrophil recruitment, and enhances the expression of IL-6 [11]. As a result of digoxin’s effects, we observe reduced IL-17 and IL-22 production, lower levels of IL-6, a trend towards reduced IL-8, and reduced neutrophil recruitment to the airways. IL-17 is a known positive regulator of mucin expression [11] and, consistent with our observed reduction in IL-17, is reduced in the lungs of digoxin treated animals. IL-17 enhances the production of IL-13 by Th2 cells [50, 51]; thus, digoxin treatment is effective in reducing IL-13 expression in the lungs of BRSV-infected calves.

## Discussion

There is currently no vaccine available to prevent RSV infection, and there are few antiviral therapies available which have proven efficacy against RSV infection in humans or animals. Corticosteroids have long been used in supportive therapy for patients with severe RSV bronchiolitis [52]. However, their efficacy is questionable, and they are nonspecific in their ability to block inflammation associated with severe viral infection. Here, we demonstrate that therapeutic inhibition of IL-17 has the capacity to limit RSV-associated disease in the neonate, without significant adverse impacts on viral burden or antiviral immunity. While digoxin is not an appropriate compound to be administered to human infants, our results suggest that a targeted approach focusing on IL-17 and Th17 immunity offers a promising strategy for therapeutic intervention in severely ill patients.

RSV replicates in the ciliated epithelial cells that line the respiratory tract. However, *in vitro*, RSV infection does not cause measurable cytopathology in primary human airway epithelial cells [27]. Instead, it is likely that the host immune response is responsible for the severity of RSV disease [26–29]. Neutrophils are the predominant cell type in the BAL of infants and animals with severe bronchiolitis [53–55]. In human infants, the peak neutrophil response appears to coincide with maximum clinical severity and viral load [56]. Given that IL-17 is a potent activator of the neutrophil response, it is logical to assume that inhibition of IL-17 and Th17 immunity may prevent exacerbated neutrophilia and thus, improve overall disease outcome. Indeed, IL-17 is elevated in tracheal aspirates from severely ill, ventilated infants compared to healthy controls [35, 37, 38]; and mice deficient in IL-17 display reduced mucus production and increased CD8 T cell responses in the lungs during primary RSV infection [35]. However, the role of IL-17 in RSV-associated disease is clearly complicated, as multiple studies in human infants have concluded that IL-17 expression negatively correlates with disease severity [30, 34, 39], suggesting that it may play a role in defense against severe RSV-associated bronchiolitis. Reports by Faber *et al*. examined nasopharyngeal aspirates during the recovery phase of the illness and found that IL-17 was increased in nonventilated infants, but not in ventilated infants, at the time of discharge [30]. However, there was no difference in IL-17 between the two groups when they were admitted to the hospital. Christiaansen *et al*. analyzed IL-17 concentrations in nasal excretions at the time of hospitalization, and observed that more severe clinical signs (either difficulty breathing and retraction) correlated with a reduced overall Th17 profile. Results from murine models suggest a dose-dependent relationship between Th17 and Th2 immunity, whereby IL-17 can both regulate and synergize with IL-13 to influence the inflammatory response in the airways [50, 51]. Thus, the effect of kinetics, concentrations and the presence of other related inflammatory molecules may explain these disparities. Our results demonstrate that therapeutic inhibition of IL-17 production results in reduced airway neutrophil recruitment and reduced RSV-associated disease. However, digoxin treatment did not fully ablate IL-17 production in the lungs of our BRSV infected calves. Thus, consistent with a possible dose-dependent effect, our results suggest that excess IL-17 may be damaging to the host, but some IL-17 production may indeed be beneficial.

Digoxin has been reported to antagonize binding of RORγt to its transcriptional activation sites [57], and to specifically inhibit IL-17 production by both γδ T cells and Th17 cells in murine and human systems [15–17, 19, 57, 58]. Ours is the first description of digoxin having the capacity to prevent IL-17 production by immune cells in another mammalian species. More importantly, our studies are the first to show that normal, therapeutic concentrations of digoxin have the capacity to inhibit IL-17 expression *in vivo* in a non-rodent species. The initial publication by Huh *et al*. reporting RORγt-specific activity of digoxin used very high doses of digoxin to block IL-17 production *in vitro* by both murine and human cells, and used similarly high doses to block *in vivo* production of IL-17 in mice. Subsequent *in vivo* studies using rodents have also used high doses of the drug [17, 18, 20–22]. However, digoxin has a narrow therapeutic range in humans and other species, with therapeutic ranges reported at 0.8–2 ng/mL in serum, and toxic levels reported at ≥4 ng/mL. Thus, high dose digoxin treatment is not practical for non-rodent species. However, the dose to effect RORγt-binding activity has not been empirically determined, and until now, it has been unknown if physiological concentrations of digoxin could inhibit IL-17 production *in vivo* in species other than mice. Cardiac glycosides have long been known to have anti-inflammatory activities [59], even at physiologic doses, suggesting that very high doses of digoxin may not be necessary to impact IL-17 production. Interestingly, a physiologic dose of digoxin (4 μg/kg/day) was shown to significantly inhibit pulmonary neutrophil and monocyte recruitment in a murine model of pneumococcal pneumonia [24, 25], resulting in significantly increased mortality in the treated animals. Although IL-17 expression was not explored in these studies, the resultant phenotype is suggestive of a possible impact on Th17 immunity. Given this evidence, we hypothesized that therapeutic ranges of digoxin may be sufficient to inhibit IL-17 production in our model of neonatal BRSV infection. Historical studies have suggested that the half-life of digoxin is very short in cattle [41] and supported the use of intravenous drug treatment, rather than oral. However, these studies were performed in adult cows, rather than preruminant calves. In preliminary dose-finding studies, we determined that oral digoxin treatment resulted in measurable serum digoxin levels. Therefore, to minimize stress to the animals, we chose an oral dosing scheme for our studies. We did not perform pharmacokinetics studies here; however, we monitored digoxin levels at a set time each day, to ensure consistent sampling. We used an initial dose of 5 μg/kg of digoxin, given twice per day, but quickly increased the dose based both upon the results of our initial pilot studies, and the results of our daily digoxin ELISA analysis on the serum. We ultimately arrived at a dose of 80 μg/kg/feeding, which achieved a relatively consistent therapeutic range of 0.8–2 ng/mL in our calves, when sampled at the designated sampling time. While this dose did not fully abrogate IL-17 expression, it significantly reduced ex vivo production of IL-17 by PBMC and BAL mononuclear cells, and reduced expression of IL-17 in the BRSV infected lung. It is probable that concentrations of digoxin varied in the tissues and serum between feedings, and future studies should perform a more thorough analysis of the drug pharmacokinetics. Furthermore, given the local nature of RSV infection, a more targeted approach of intranasal or aerosol digoxin treatment may further minimize the risk of the drug. Importantly, however, the ultimate goal of our studies is not to support the use of digoxin as a therapeutic agent for use against RSV, but instead, to highlight the importance of IL-17 and the Th17 immune response as a potential target for future development of small molecule or antibody-based therapies in human and animal patients with severe RSV disease. This is an area of active investigation, and several other small-molecule inhibitors of the RORγt transcription factor have been identified, including ursolic acid [60, 61] and the synthetic small molecules GSK805 [57] and TMP778 [57, 62, 63]. These compounds are currently cost prohibitive for clinical use. However, based upon our results, their activity should be further explored for reducing RSV-associated disease, as should strategies for the production of more cost-effective IL-17 targeting strategies.

A limitation of our study for interpretation towards therapy is that digoxin administration was initiated prior to infection and continued through the end of the study, rather than initiated following the appearance of clinical signs. Results from Huh *et al*. and others [15, 17, 18, 20–23, 57] suggest that digoxin treatment is efficacious in preventing inflammation even after establishment of chronic diseases. It is unknown if blockade of IL-17 after appearance of clinical signs will have any effect on disease progression during an acute inflammatory response, such as during RSV infection, but remains an important question. However, the objective of our work was two-fold: 1) to determine the importance of IL-17 during BRSV infection; and 2) to determine if targeting IL-17 can be used as a possible treatment avenue. γδ T cells have the capacity to serve as an early source of IL-17, and CD4 T cell differentiation begins to occur in the lymph nodes within the first few days of infection [11]. To properly address the first point, we chose to initiate treatment prior to BRSV inoculation to ensure that digoxin was acting on both early γδ T cell responses and in the initial, critical stages of T cell priming in the LN. Furthermore, we reasoned that if the drug failed to improve disease outcome when given so early in the infection, our hypothesis would be thoroughly disproven and would negate the need for further investigation into the ideal therapeutic window and the appropriate dose range for inhibiting IL-17 production.

An unexpected effect of digoxin treatment was the enhanced production of IgA in the nasal fluid. IL-17 is generally linked to enhanced IgA production [11]; thus, the reduced production of IL-17 during digoxin treatment could presumably negatively impact IgA production. However, as shown in Figs 7 and 8, digoxin treatment had significant downstream effects on the inflammatory environment in the mucosa, including changes in expression of the mucin genes, IL-6, IL-22, and IL-13; as well as other possible changes that were not assessed. In other models, neutrophil infiltration and increased levels of IL-8 have been shown to negatively correlate with mucosal IgA production [64, 65], and neutrophils are known to inhibit B cell class-switching and antibody production [reviewed in [66]]. Thus, it is conceivable that alterations in the inflammatory milieu, or reduced in neutrophil recruitment, resulted in accelerated or more efficient IgA secretion.

In conclusion, our results suggest that exacerbated IL-17 and Th17 immunity plays a pathogenic role during RSV infection in the neonate; and that inhibiting IL-17 production has a positive impact on RSV-associated disease. Digoxin therapy does not fully abrogate *in vivo* production of IL-17; thus, it remains unclear if some degree of Th17 immunity plays a beneficial role in host defense. Our results support the further development of targeted immunotherapies to modulate the host inflammatory response during RSV infection, and suggest that the neonatal calf model represents a valuable model for preclinical testing of such novel intervention strategies.
